# New insights towards strikingly improved room temperature ethanol sensing properties of p-type Ce-doped SnO_2_ sensors

**DOI:** 10.1038/s41598-018-26504-3

**Published:** 2018-05-24

**Authors:** Manjeet Kumar, Vishwa Bhatt, A. C. Abhyankar, Joondong Kim, Akshay Kumar, Sagar H. Patil, Ju-Hyung Yun

**Affiliations:** 1 Department of Electrical Engineering, Incheon National University, Incheon, 406772 South Korea; 2 Department of Materials Engineering, Defence Institute of Advanced Technology, Girinagar, Pune 411025 India; 3 Department of Nanotechnology, Sri Guru Granth Sahib World University, Fatehgarh Sahib, 140 407 Punjab India; 4 Center for material characterization, CSIR-National chemical laboratory, Pashan, Pune 411008 India

## Abstract

In this article, room temperature ethanol sensing behavior of p-type Ce doped SnO_2_ nanostructures are investigated successfully. Interestingly, it is examined that the abnormal n to p-type transition behavior is caused by Ce doping in SnO_2_ lattice. In p-type Ce doped SnO_2_, Ce ion substituting the Sn is in favor of generating excess holes as oxygen vacancies, which is associated with the improved sensing performance. Although, p-type SnO_2_ is one of the important materials for practical applications, it is less studied as compared to n-type SnO_2_. Pure and Ce doped SnO_2_ nanostructures were successfully synthesized by chemical co-precipitation method. The structure, surface morphology, unpaired electrons (such as free radicals), and chemical composition of obtained nanoparticles were studied by various kinds of characterization techniques. The 9% Ce doped SnO_2_ sensors exhibit maximum sensor response of ~382 for 400 ppm of ethanol exposure with fast response time of ~5 to 25 sec respectively. Moreover, it is quite interesting that such enhancement of ethanol sensing is unveiled at room temperature, which plays a key role in the quest for better ethanol sensors. These remarkably improved sensing results are attributed to uniformly distributed nanoparticles, lattice strain, complex defect chemistry and presence of large number of unpaired electrons on the surface.

## Introduction

In past six decades, metal oxide semiconductors (MOS) have been one of the most prevailing gas sensing materials among large miscellany of materials on the grounds of its low production cost, tranquility during use and prominent selectivity for various gases in addition to high detection^1–5^. Over the past few decades, an intense requirement of gas sensors has become progressively greater by virtue of elevated atmospheric pollution leading towards the emission of hazardous gases^6–10^. One of the vital requirements of such gas sensors can be ethanol sensors. Those working on ethanol synthesis, experience hardship from the huge probability of being victims of digestive track cancer and increased risk of respiratory symptoms of a threshold limit (1000 ppm). Thus, an increasing demands and emerging challenges for detection of ethanol gas at ppm/ppb level have enforced the researchers to fabricate low cost, high performance and relatively stable ethanol sensors. With the growing interest in progression of sensing devices, more and more exhilarating results of MOS have been reported in literature^10–14^. The gas sensors developed by various types of MOS exhibited good sensitivity to ethanol at different operating temperatures (OT)^15–19^ suggesting that most of the ethanol sensors work at higher temperature. Various MOS such as TiO_2_, GeO_2_, Cr_2_O_3_, Mn_2_O_3_, NiO, CuO, CdO, CeO_2_, MgO, BaO, In_2_O_3_, WO_3_, V_2_O_3_, Fe_2_O_3_, Nb_2_O_5_, MoO_3_, Co_3_O_4_, Ta_2_O_5_, La_2_O_3_, Nd_2_O_3_ etc. are well-known and widely used for metal oxide based sensors for the detection of different chemical inputs^20,21^.

Among them, pure SnO_2_ is one of the most prominent n-type, wide band gap (3.6 eV) MOS with a carrier concentration of 5.7 × 10^20^ cm^−3^ making it an outstanding material for chemical gas sensors^22–24^. Although pure SnO_2_ has an excellent sensitivity but the major drawbacks are its poor selectivity which hinders a lot to meet up all practical key parameters such as high sensitivity, faster response-recovery time, stability and low power consumption for realistic sensor applications^20,25–28^. Thus, despite of substantial research work carried out on sensing materials, still there is a scope of exploration to achieve higher sensitivity and better selectivity for hazardous gases at room temperature (RT). For that, metal ion doping in SnO_2_ lattice may be one of the influencing factors for the furtherance in sensing properties of SnO_2_^29^. In case of doped SnO_2_, substitutional and interstitial point defects may exist depending on the rearrangement of the internal defects in SnO_2_ lattice^30^. In addition, the semiconducting behavior can be predicted depending on the type of doping (donor or acceptor) introduced to the host material in order to tailor the band bending and band alignments on which the amount of gas sensing enhancement relies. It is noteworthy that gas sensing has been explored using SnO_2_ exhibiting n-type behavior but their equivalent p-type counterpart was unexpectedly absent for a long time. In past a decade, the exploration of novel p-type SnO_2_ for gas sensors has been started^31–33^ and the researchers and technologists around the globe are making an effort to discover an effective method to attain the p-type conduction in the n-type SnO_2_. In literature, p-type conductivity in SnO_2_ has been achieved by doping M_Sn_ metal ions (where, M = Li, Al, Ga, In and Cd) acting as a substitutional dopant in SnO_2_ playing an important role to enhance the gas sensing characteristics^30,34,35^. Hence, the understanding of synthesis and fabrication of p-type SnO_2_ gas sensors would be a worthy research area to open up several new possibilities in device applications.

In this study, authors have used the state of art to explore p-type conduction mechanism in Ce-doped SnO_2_ to characterize RT ethanol sensing. Although Ce-doped n-type SnO_2_ based sensors have already been used for the detection of ethanol but reported operating temperatures of these sensors are as high as 300 to 500 °C^21,27,36^ and no reports have been shown on p-type conduction in Ce-doped SnO_2_ ethanol sensors at RT. Therefore, the fabrication of stable and highly selective gas sensing materials working at RT is still highly desirable. Present work demonstrated the RT ethanol sensors based on p-type Ce-doped SnO_2_ nanostructured devices to understand their mechanism under consideration of several aspects that influence the sensor performance including defect chemistry, surface morphology, several diffusion processes, and associated unpaired electrons in the form of free radicals. However, from the literature and present study, authors realized that the level of understanding of these mechanisms is still lacking and in-depth research in this area is required. To the best of our knowledge, it is the first systematic report on p-type Ce doped SnO_2_ nanostructure based RT ethanol sensor. The results described in this article indicates that 9 wt % Ce doped SnO_2_ sensor is highly sensitive and selective towards ethanol detection at RT.

## Results

### XRD analysis

**Figure 1:**
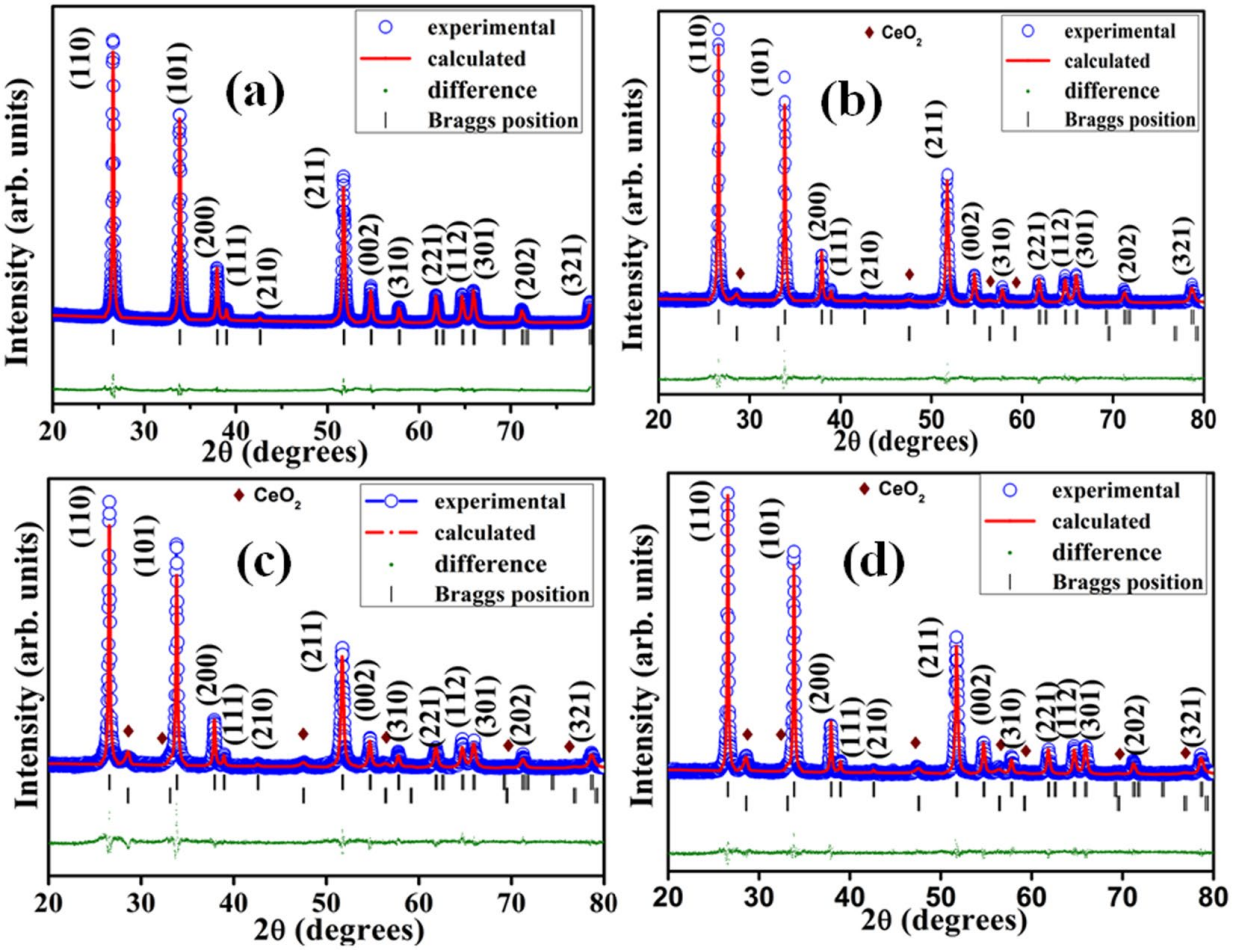
Rietveld fit of pure SnO_2_ and Ce doped SnO_2_ nanomaterial with addition of different concentrations of Ce: (**a**) 0%, (**b**) 3%, (**c**) 6% and (**d**) 9%.

The XRD patterns of pure and Ce doped SnO_2_ nanostructured samples are elucidated in Fig. 1. As it can be seen that, pure SnO_2_ have tetragonal rutile structure which is indexed with JCPDS file 00-041-1445^37^. It has been observed from Fig. 1(b–d) that Ce doped SnO_2_ nanostructured samples have all the peaks of SnO_2_ with a few peaks of CeO_2_. These peaks were indexed with the JCPDS files of SnO_2_ and CeO_2_ (JCPDS file no.: 01-075-0120)^38^. The XRD pattern of pure and Ce doped SnO_2_ nanomaterials have been refined by Rietveld refinement using FULLPROF Suite programme. The background for all samples was refined by 6^th^ degree Polynomial function. The pseudo-voigt profile function (linear combination of Gaussian and Lorentzian) was used to describe the peak shape of all the samples. The Rietveld refinement data of pure and Ce doped SnO_2_ nanomaterial samples are summarized in Table 1. It can be seen from Fig. 1 that the diffraction peaks of all the samples were well fitted. The lattice parameters, atomic position and phase fraction are listed in Table 1. The increase in magnitude of lattice parameter and cell volume of Ce doped SnO_2_ are shown in Table 1. The lattice parameter ‘a’ and ‘c’ of the Ce doped SnO_2_ unit cell is found to increase linearly with the Ce concentration following Vegards law^39,40^ which is shown in figure (see Supporting Information, Fig. S1). It is also observed that the full width at half maxima (FWHM) increases with an increase in Ce content in the SnO_2_. Hence, this indicates that Ce doping in the SnO_2_ lattice leads to decrease in crystallite size. From the increased lattice constant with the addition of Ce content, it is confirmed that Ce ions have been effectively doped in SnO_2_ lattice and some part is present in the form of CeO_2_^36,41^. The substitution of Sn by Ce creates vacancies and disturb local arrangements of the crystal structure which is further confirmed from ESR analysis. Similar observations have been reported in the literature by Yang *et al*.^42^. The slight deviation in atomic position has been observed that may induce lattice strain which is further confirmed from the Williamson-Hall plot^43^ (see Supporting Information, Fig. S2). The value of average micro-strain for pure SnO_2_ is 1.28 × 10^−3^ and found to be increased in case of 9% Ce doped SnO_2_ nanomaterial which is shown in Table 1. It shows that with the increased Ce content in SnO_2_, the little deviation in atomic position is found and lattice constant also increases with an increase in micro strain values from 1.28 × 10^−3^ to 3.4 × 10^−3^. Due to incorporation of Ce ions into the SnO_2_ lattice, this causes increase in lattice parameter and micro strain. The influences of increased lattice parameter and micro strain on sensing characteristics have been discussed at the end of this section. The surface morphology and elemental composition of the sensor materials have been studied using FESEM and EDS measurement (see Supporting Information, Figs S3 and S4).

**Table 1:** Atomic position, Phase fraction, Lattice parameter, Volume, χ^2^ values obtained by Rietveld refinement and strain value using W-H analysis.

Samples wt.% of Ce	Atomic position	x	y	z	Phase Fraction (%)		*χ* ^2^	Lattice Parameter		Volume (Å)^3^	Strain
								*a* (*Å*)	*c* (*Å*)		
0	Sn	0.00	0.00	0.00	SnO_2_	100	1.96	4.741	3.188	71.656	1.28 × 10^−3^
	O	0.301	0.301	0.00							
3	Sn	0.00	0.00	0.00	SnO_2_	93.23	1.70	4.742	3.189	71.709	2.3 × 10^−3^
	O	0.30	0.30	0.00	CeO_2_	6.77					
6	Sn	0.00	0.00	0.00	SnO_2_	90.68	2.08	4.743	3.190	71.762	3.2 × 10^−3^
	O	0.299	0.299	0.00	CeO_2_	9.32					
9	Sn	0.00	0.00	0.00	SnO_2_	85.85	2.3	4.744	3.191	71.815	3.4 × 10^−3^
	O	0.298	0.298	0.00	CeO_2_	14.15					

### TEM and HRTEM analysis

**Figure 2:**
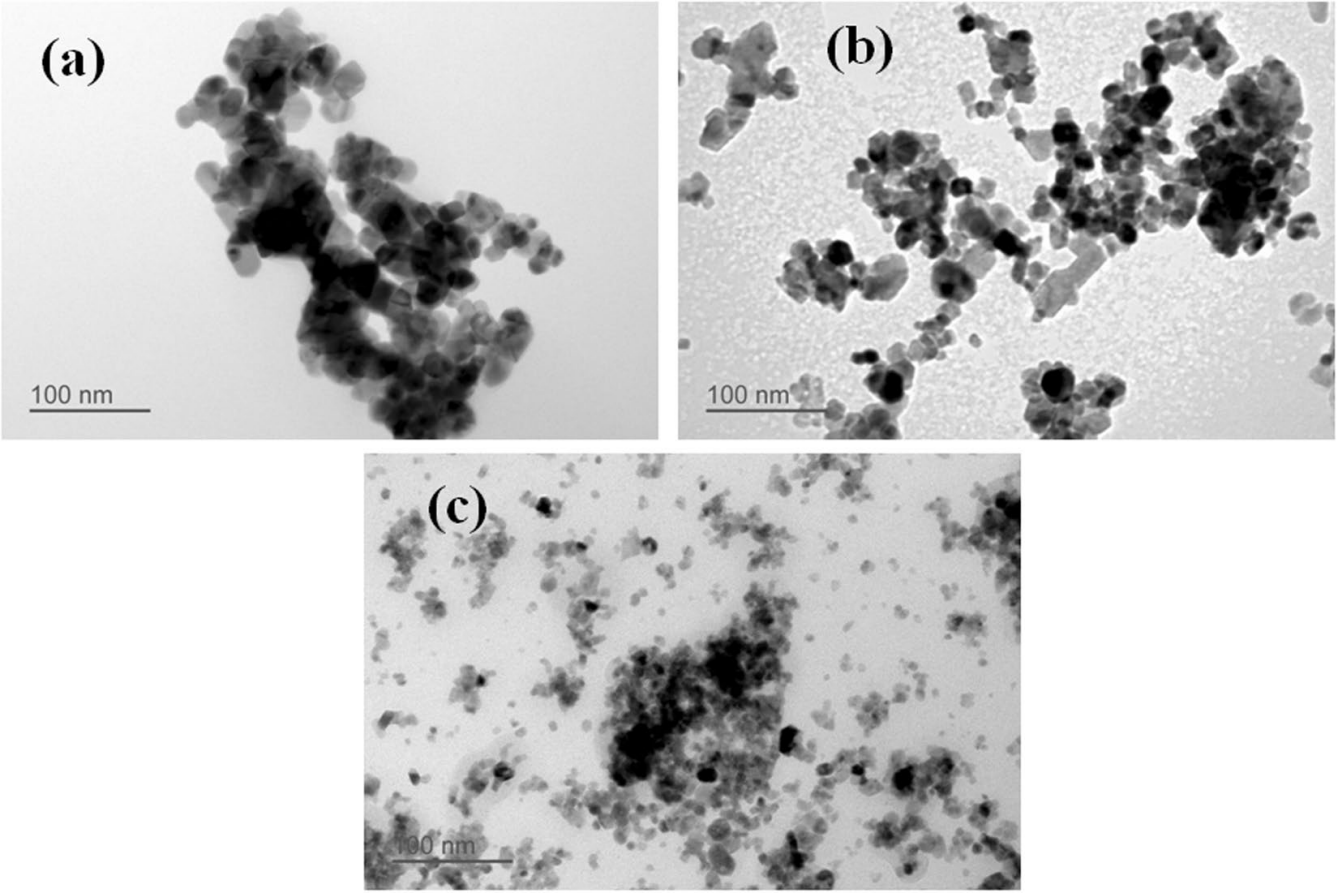
TEM micrograph of pure and Ce doped SnO_2_ with addition of different concentrations of Ce: (**a**) 0%, (**b**) 6% and (**c**) 9%.

**Figure 3:**
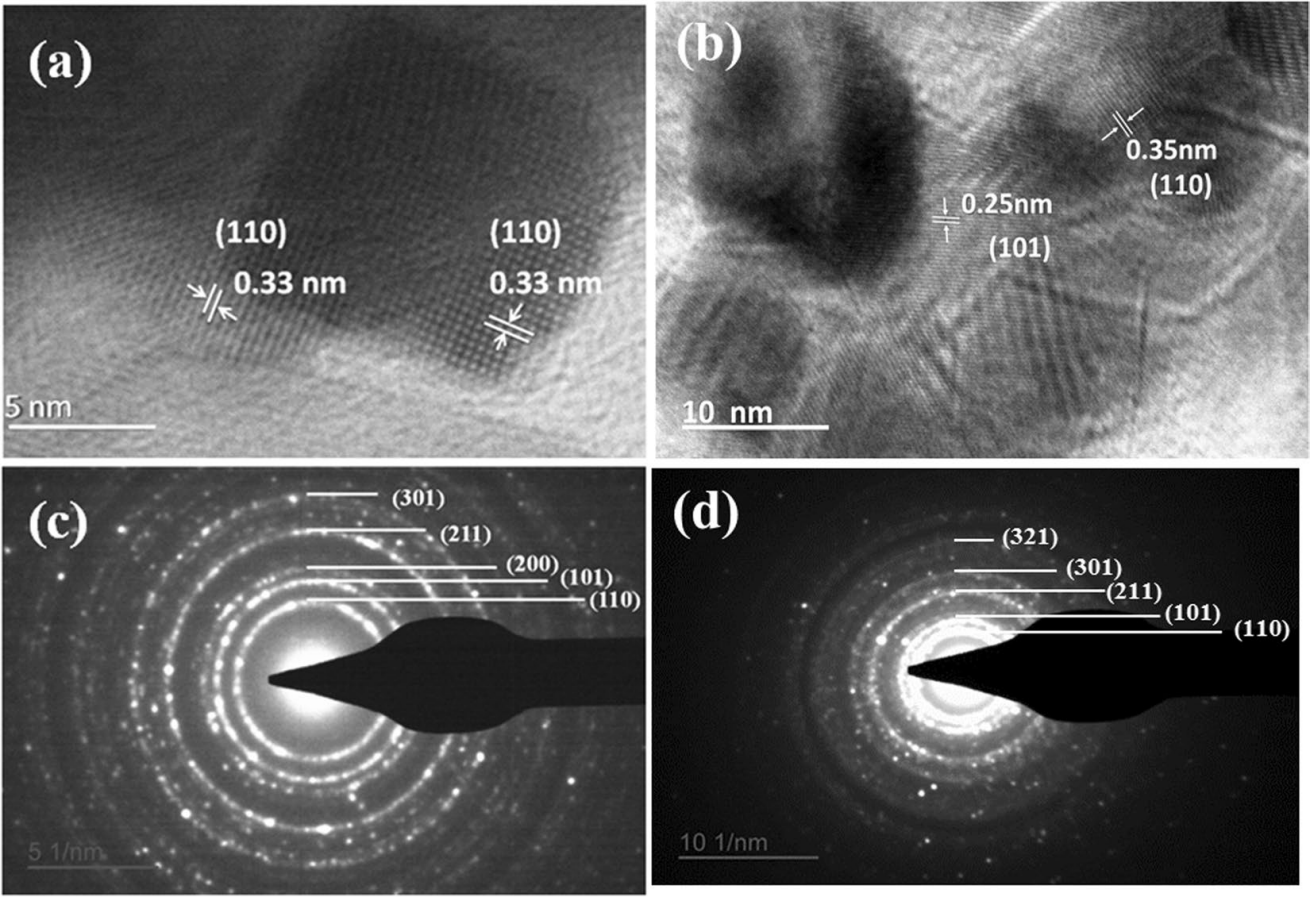
HRTEM micrograph of (**a**) pure SnO_2_ (**b**) 9% Ce doped SnO_2_ samples. SAD pattern of the (**c**) pure SnO_2_ (**d**) 9% Ce doped SnO_2_ samples.

Figure 2(a–c) shows the TEM images of pure and Ce doped SnO_2_ nanomaterials. From TEM images, it is clear that the particle size is decreasing with the addition of Ce content. The nanoparticles are random/polygon in shape with the average particle size of ~16 nm, ~12 nm and ~6 nm for Pure SnO_2_, and Ce doped SnO_2_ (0, 6 and 9%) respectively. 9% Ce doped SnO_2_ nanomaterial results in formation of uniformly distributed nanoparticles of approximately ~6 nm particle size, which is twice of the Debye length for SnO_2_. The Fig. 3(a,b) shows the HRTEM images of pure and Ce doped SnO_2_ nanomaterials where we can clearly see the lattice resolution of individual nanoparticles. The distance between the lattice planes for pure SnO_2_ is found to be 0.33 nm which is associated to the (110) plane of tetragonal SnO_2_. The marked lattice fringes of 9% Ce doped SnO_2_ sample is found to be 0.35 nm and 0.25 nm corresponding to (110) and (101) planes of tetragonal SnO_2_ respectively. The selected area diffraction (SAD) patterns recorded for pure and Ce doped SnO_2_ nanomaterials are shown in Fig. 3(c,d) which clearly reveal the polycrystalline nature of pure and Ce doped SnO_2_. The diffraction rings of pure SnO_2_ are indexed as (110), (101), (200), (211) and (301) confirming the tetragonal rutile structure. SAD pattern acquired for 9% Ce doped SnO_2_ also confirms the formation of tetragonal rutile structure. These results are consistent with the above mentioned XRD results.

### ESR analysis

**Figure 4:**
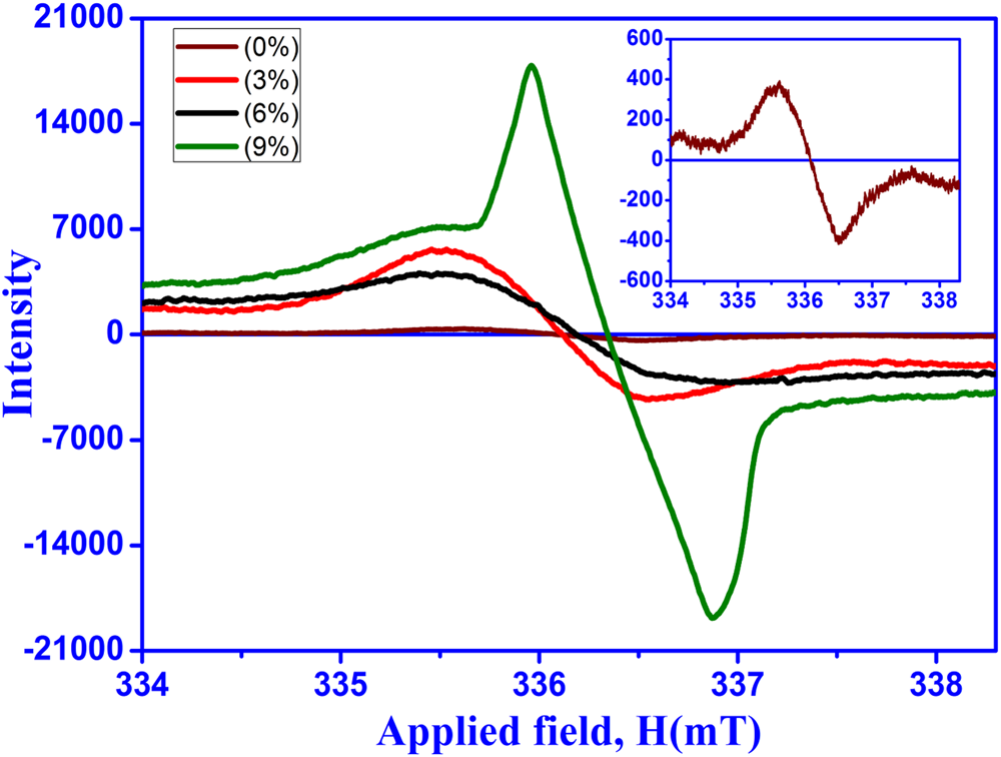
ESR spectra of pure and Ce doped SnO_2_ with addition of different concentrations of Ce: (**a**) 0%, (**b**) 3%, (**c**) 6% and (**d**) 9%. Inset shows the ESR spectra of pure SnO_2_.

The presence of unpaired electrons in the form of free radicals and charge carriers has been studied using ESR spectroscopy. The Fig. 4 shows the ESR dispersion derivative (dp/dH) as a function of magnetic field. The ESR measurements were performed at microwave frequency of ~9.45 GHz (X-band). When the external magnetic field is applied, the spin interactions of the unpaired electrons can be studied from ESR spectra recorded for pure and Ce doped SnO_2_ at RT. As it is known that the spin quantum number (s) of ½ with the magnetic moments consisting of two magnetic components ms = +½ and −½ in parallel and antiparallel respectively. These magnetic components are aligned to the applied field with the strength of B_o_ having a specific energy. There is a possibility of either absorption or emission of electromagnetic radiation by unpaired electrons and they also consist of two energy levels between which the unpaired electrons can move. The change in the two energy levels of an electron can be obtained by Δ*E* = *gμ*_*B*_*B*_0_, where, g and *μ*_*B*_ are electron’s g factor and Bohr magnetron (*μ*_*B*_ = 9.274 × 10^−24^ *JT*^−1^) respectively. ESR spectra show a resonance at 336.07, 336.1, 336.2 and 336.4 mT for pure and Ce doped SnO_2_ (0, 3, 6 and 9%) samples respectively. Although resonance field is not affected much, Fig. 4 shows that peak to peak line width and power absorption during resonance increases remarkably upon Ce addition. Small ∆Hpp and high intensity observed in 9% Ce doped SnO_2_ may be attributed to the existence of large number of intrinsic oxygen vacancies. The g-value for all samples were calculated from resonance magnetic field as shown in Table 2, which is nearly equal to the g–value of (2.008) of free electron^44^. The g-factor was calculated as $g=h\vartheta /{\mu }_{B}{B}_{0}$ similar to the reported in literature^45^. The width of the ESR signal was found to be the lowest (~0.92) in 9% Ce doped SnO_2_ sample. The narrowing of ESR line is attributed to strong exchange of unpaired electrons and delocalization of unpaired electrons over a large system of conjugated chemical bonds^46^. Thus, the defect chemistry of Ce-doped samples were characterized by studying the presence of free electrons using ESR spectroscopy and the analysis suggested that Ce doping in SnO_2_ create a huge amount of free electrons which is certainly responsible for improved sensitivity.

**Table 2:** ESR data of pure and Ce doped SnO_2_ samples.

Samples	g-factor	∆H_pp_ (mT)
Pure SnO_2_	2.009	1.32
3 wt.% Ce doped SnO_2_	2.009	1.26
6 wt.% Ce doped SnO_2_	2.007	1.46
9 wt.% Ce doped SnO_2_	2.008	0.92

### XPS analysis

**Figure 5:**
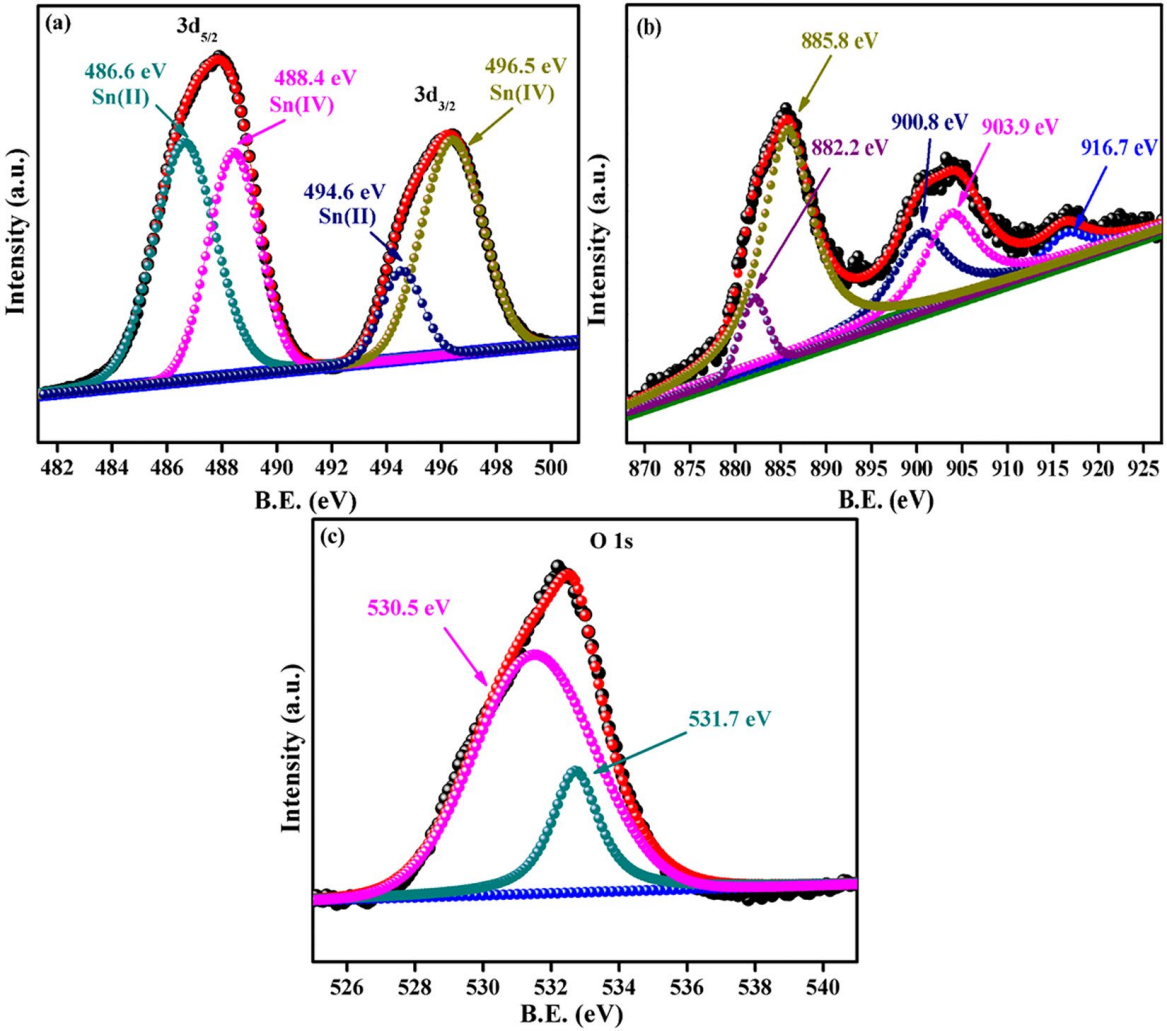
XPS core level spectra of (**a**) Sn 3d (**b**) Ce 3d and (**c**) O 1 s for 9 wt.% Ce doped SnO_2_ sample.

The core level x-ray photoelectron spectra’s of 9 wt.% Ce doped SnO_2_ sample has been investigated in vacuum of the order of 2.4 × 10^−9^ torr. All obtained peaks were fitted by the coalition of Lorentzian and Gaussian function to reduce the least square error of the fit. A linear and tougaard background function was used to account the inelastic background in the XPS spectrum. All the XPS spectra shown in Fig. 5, are referenced to C 1 s peak (284.6 eV). The deconvoluted XPS core level Sn 3d spectra’s of 9 wt.% Ce doped SnO_2_ sample indicates the presence of Sn with two oxidation state i.e Sn^2+^ and Sn^4+^ respectively shown in Fig. 5(a)^47–50^. Figure 5(b) shows the deconvoluted XPS core level Ce 3d spectra of 9 wt.% Ce doped SnO_2_ sample. The valence state of Ce 3d was determined by deconvolation of Ce 3d spectra. The Ce 3d spectra is fitted with the five subpeaks. These subpeaks corresponding to Ce^3+^ and Ce^4+^ valance state respectively. In Fig. 5(b), binding energy at 882.2 eV, 900.8 eV and 916.7 eV sub peaks were assigned to Ce^4+^ valance state^51–53^, whereas 885.8 eV and 903.9 eV sub peaks were attributed to Ce^3+^ valence state^54,55^. Above results confirmed that 9 wt.% Ce doped SnO_2_ sample displayed the presence of mixed valence state of Ce^3+^ and Ce^4+^ with the relative percentage of 70% and 30% respectively. This suggest that the content of Ce^3+^ is higher than Ce^4+^ valence state, which indicates that Ce^3+^ ion were substituted into SnO_2_ lattice. Hence Ce^3+^ substitution at Sn^4+^ site is a firm reason for n to p-type transition by Ce doping in SnO_2_ lattice which is further confirmed from Hall measurement results (see Supporting Information, Table S1). The substitution at Sn^4+^ by Ce^3+^ triggered the creation of oxygen vacancy defects in SnO_2_ lattice, which is well matched with ESR results. The O 1 s spectrum for 9 wt.% Ce doped SnO_2_ presented in the Fig. 5(c). It has been observed from the Fig. 5(c), that two components are present at 530.5 eV and 531.7 eV. This first peak (530.5 eV) is associated to lattice oxygen bound to Sn^56,57^ and second peak at 531.7 eV corresponds to oxygen deficient region in SnO_2_ matrix^58,59^.

### Sensing measurements

**Figure 6:**
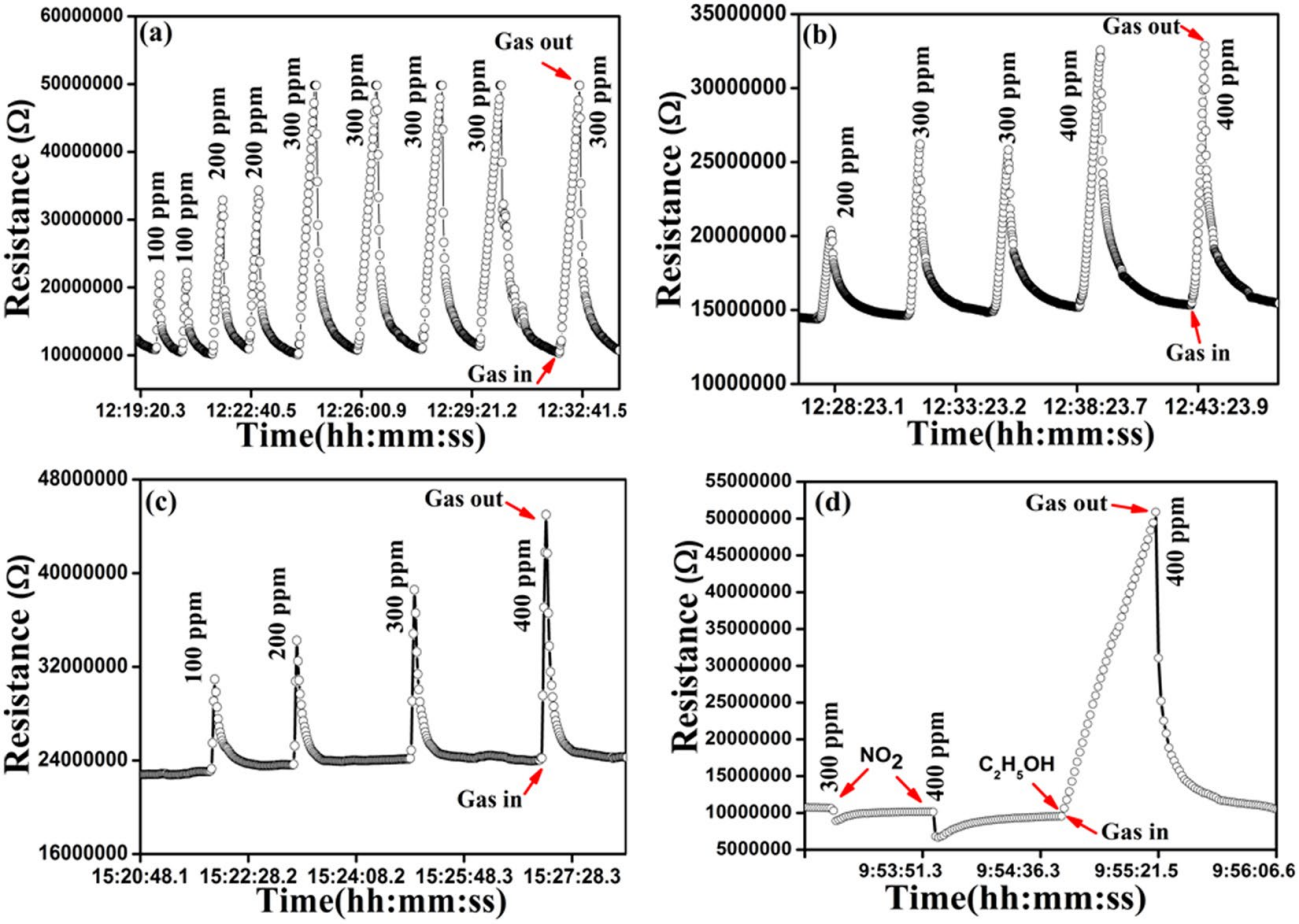
(**a**) Reproducibility of 9% Ce doped SnO_2_ sensor response for 200, 300 & 400 ppm of ethanol (**b**) Changes in sensing response exhibited for 200, 300 and 400 ppm of ethanol by 6% Ce doped SnO_2_ sensor and (**c**) Response of 3% Ce doped SnO_2_ sensor for 100, 200, 300 & 400 ppm of ethanol (**d**) Response of 9% Ce doped SnO_2_ sensor for NO_2_ and ethanol.

**Figure 7:**
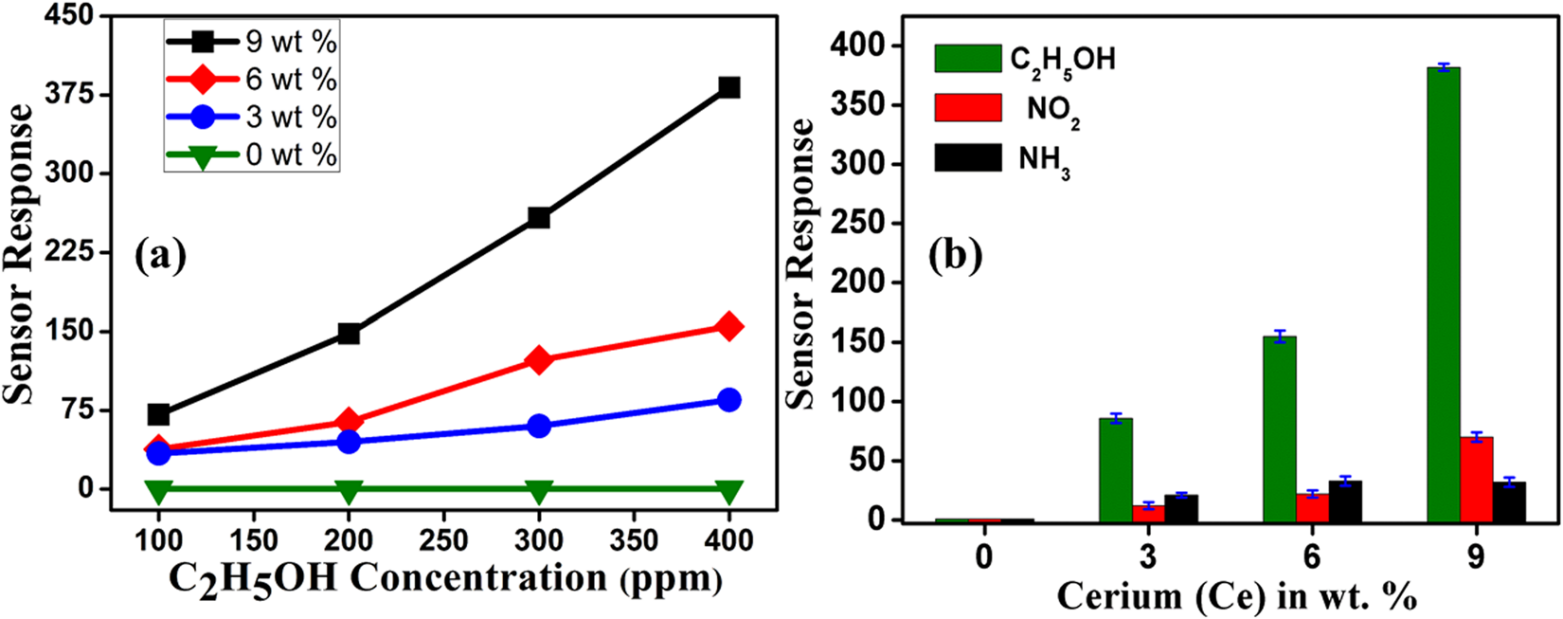
(**a**) Linearity of sensor response shown over concentration of 100, 200, 300 and 400 ppm ethanol for pure SnO_2_ and Ce doped SnO_2_ sensor (**b**) Sensor response of pure and Ce doped SnO_2_ sensor as a function of Ce concentrations at 400 ppm of each C_2_H_5_OH, NO_2_ and NH_3_ gas.

The gas sensing measurements of pure and Ce doped SnO_2_ sensors were performed at RT for ethanol detection. Before starting the measurements, each sensor was initially purged with synthetic air for 10 minutes to stabilise the base line resistance. The exposure time to the chemical inputs was maintained to be 5 sec and for recovery, the exposure to reference synthetic air was maintained to 10 sec respectively. The resistance of sensing materials increased or decreased to the exposure and removal of chemical inputs. The gas sensitivity is defined as follows;

1 $$\begin{array}{cc}{S}_{R}=\frac{{R}_{g}-{R}_{a}}{{R}_{a}}\times 100 & ({\rm{For}}\,{\rm{reducing}}\,{\rm{gas}})\end{array}$$

2 $$\begin{array}{cc}{S}_{O}=\frac{{R}_{a}-{R}_{g}}{{R}_{g}}\times 100 & ({\rm{For}}\,{\rm{oxidizing}}\,{\rm{gas}})\end{array}$$

where, Rg is the electrical resistance in presence of gas and Ra is the electrical resistance in clean synthetic air atmosphere only. Figure 6(a,b) shows the repeatable operation cycles of 9 and 6% Ce doped SnO_2_ sensors in the ethanol exposure at RT. The response signal of 9, 6 and 3% Ce doped SnO_2_ sensors for various amount of concentration at RT is shown in Fig. 6(a–c). Figure 6(d) shows the response signal of 9% Ce doped SnO_2_ sensors for NO_2_ and ethanol exposure. It has also been observed that sensor response (recovery) times are of the order of ~5–30 secs (30–60 secs) as seen from Fig. 6(a–d). The sensor response of pure and Ce doped SnO_2_ as a function of ethanol concentration is shown in Fig. 7(a). It is evident from the Fig. that 9% Ce doped SnO_2_ sensor exhibits higher sensitivity for various amount of ethanol concentration at RT. The 9% Ce doped SnO_2_ sensor shows a striking improvement of RT ethanol sensing behaviour as compared to pure SnO_2_. Selectivity behaviour of the pure and Ce doped SnO_2_ sensor was tested with 400 ppm of C_2_H_5_OH (ethanol), NH_3_ (ammonia) and NO_2_ gas.

The sensitivity values for all the above-mentioned chemical inputs are shown in Fig. 7(b). We can clearly observe from the Fig. 7(b) that 9% Ce doped SnO_2_ sensor is highly selective to ethanol gas at RT. The use of the sensing device for longer duration with accuracy depends upon reproducibility, reliability and repeatability. The sensor performance should not vary with time and operation cycles. The stability of 9% Ce doped SnO_2_ sensor towards ethanol at RT has been studied for 180 days. It has been confirmed that 9% Ce doped SnO_2_ sensor displays better stability and repeatability even at the end of 6^th^ month which is ~97–98% as compared to initial measurements (see Supporting Information, Fig. S5). Such stability and repeatability analysis suggest that the 9% Ce doped SnO_2_ sensor can be used for long duration, which is one of the major advantages for practical applications.

## Discussion

**Figure 8:**
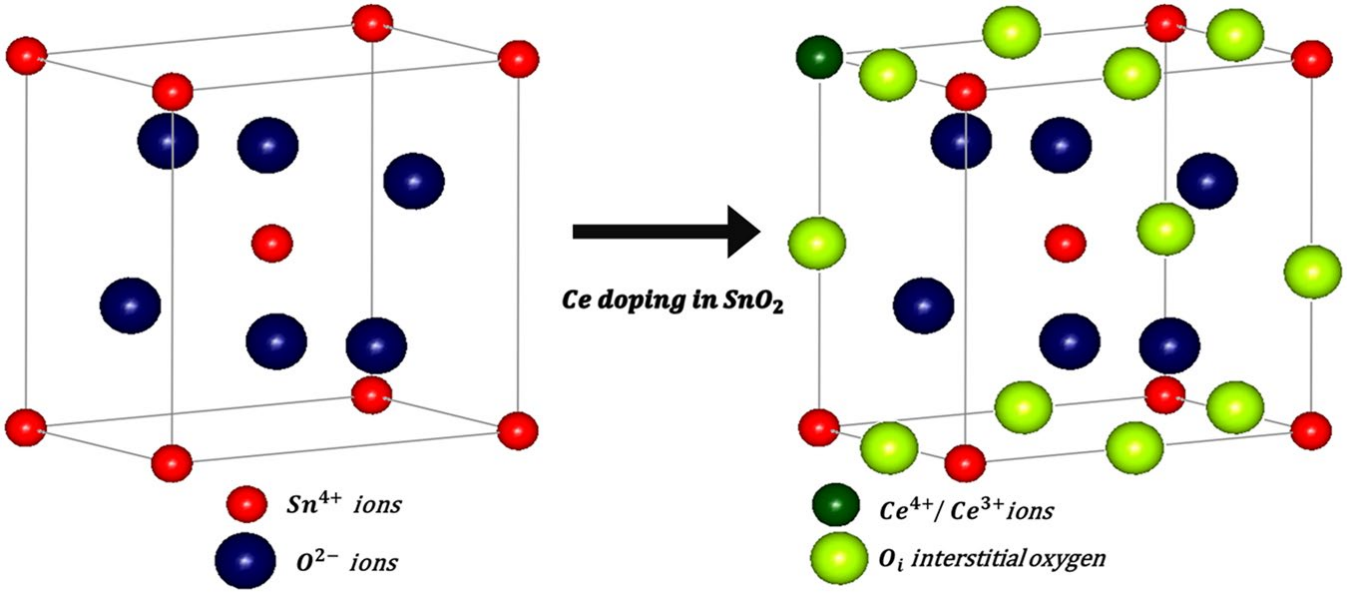
Schematic illustration of effect of Ce doping in SnO_2_ lattice.

As it is clear that the undoped SnO_2_ will never exhibit p-type semiconducting behaviour. In order to overcome the barriers regarding the enhancement of the gas sensing, different amount of Ce has been doped into pure n-type SnO_2_ and sensing measurements of p-type Ce doped SnO_2_ has been studied systematically. As shown in Fig. 7(a), a linear sensor response has been observed for C_2_H_5_O_5_ concentration (ppm) varying from 100 to 400 ppm indicating enhanced sensing for 9 wt% Ce doped SnO_2_ material at RT. As discussed from XRD measurements, it is evident that Ce has been incorporated in SnO_2_ lattice which causes increase in lattice parameter and micro strain. From the above analysis, it is confirmed that defect chemistry has been modified with the incorporation of Ce into SnO_2_. The assumptions regarding defect states can be made in order to identify the type of defects induced in the structure with doping: (i) substitution of Ce in place of Sn (Ce_Sn_) (ii) interstitial oxygen (O_i_) formation because of the occupation of Sn position by Ce atom and (iii) a complex defect (Ce_Sn_ + 2O_i_) may be present at the Sn site. Figure 8 shows the schematic illustration of defect chemistry caused by Ce doping in SnO_2_ lattice. In order to justify the drastically enhanced sensing mechanism in Ce doped SnO_2_, one needs to identify the dominance of the defects as mentioned above and an in-depth analysis is required. As discussed earlier from the ESR measurements, it is clear that there is a strong exchange of unpaired electrons and delocalized unpaired electrons in the form of free radicals. Small ∆Hpp and high intensity is observed in 9% Ce-doped SnO_2_ may be attributed to the existence of large number of intrinsic oxygen vacancies. This suggested that the increase in the Ce doping concentrations leads to the increase in the interstitial oxygen atoms present in the structure/system. This is because there is a reduction in the Sn content via substitution of Ce (Ce_Sn_) which is also evident from EDS analysis (see Supporting Information, Fig. S4). Hence, it is revealed that the doping conditions have been resulted in Sn-poor/O-rich condition which should be the reason for p-type semiconductor^34,60^. From the literature, it is confirmed that the Ce_Sn_ acts as a donor because of the lower formation energy. In addition, the complex defect (Ce_Sn_ + 2O_i_) present at the Sn site consists of relatively high formation energy and behaves as an acceptor. It is here to be understood that there is a chance of existence of self-compensation between lower formations energized donors and acceptors respectively. Such self-compensation will suppress Ce_Sn_ by (Ce_Sn_ + 2O_i_) complex defect and can be predicted to behave like an acceptor concluding that the Ce doped SnO_2_ behaves as a p-type semiconductor. It has been confirmed that, when C_2_H_5_OH gas exposed to all the sensors, the 9% Ce-doped SnO_2_ sensor shows the highest response (SR = 382) for 100 to 400 ppm concentrations of C_2_H_5_OH at RT. The improved sensors response of the p-type Ce-doped SnO_2_ nanostructures to ethanol at RT can be studied in more detail in order to clarify sensing mechanism. The defect of the p-type Ce-doped SnO_2_ and their sensing mechanism can be explained using the following reactions 3, 4 and 5 (in Kröger-Vink notation)^30,61^.

3 $$Sn{O}_{2}\to 2C{e}_{Sn}+3{O}_{0}^{X}+{V}_{0}^{\cdot \cdot }$$

4 $$\frac{1}{2}{O}_{2(g)}\leftrightarrow {O}_{(ads)}^{-}+{h}^{\cdot }$$

However, Ce_Sn_ is the Ce substitution in Sn sites and ${O}_{0}^{X}$ is the lattice oxygen and ${V}_{0}^{\cdot \cdot }\,$ denotes the two positively charged vacancies on an oxygen sites. Thus, in p-type Ce-doped SnO_2_, the adsorption of negatively charged oxygen ion can produce the holes for conduction which is shown in the reaction-4^62^. The interactions between ethanol and surface adsorbed oxygen in p-type Ce-doped SnO_2_ can be described by reaction 5. The resulting gas-sensing equation may be considered according to the charges of the adsorbed oxygen ions under the hypothesis of complete oxidation of C_2_H_5_OH.

5 $${C}_{2}{H}_{5}O{H}_{(g)}+6{O}_{(ads)}^{-}+6{h}^{\cdot }\to 2C{O}_{2(g)}+3{H}_{2}{O}_{(g)}$$

Thus, complete oxidation of C_2_H_5_OH in the above reactions increased the resistance and concurrently decreases the conductance of the surface region of p-type Ce-doped SnO_2_ upon exposure to ethanol. Such changes in resistance while exposure to ethanol in Ce-doped sensors shown in Fig. 6.

In order to summarize the improved gas sensing behaviour of 9 wt % Ce doped SnO_2_ sensor towards ethanol at RT, four major factors are responsible (i) more structural defects present in the sample, (ii) particle size becomes twice of the Debye length and (iii) high surface to volume ratio and (iv) presence of a large number of unpaired electrons on the sensor surface. It can therefore, be inferred from XRD analysis that Ce doping in SnO_2_ leads to an increase in lattice strain as well as deviation from atomic position of the host material observed. This deviation from atomic position of the host material and increased lattice strain will result in the formation of more structural defects within the system. When Sn is substituted by isovalent Ce atoms, Ce^4+^ could be easily reduced to Ce^3+^ which leads to the formation of an acceptor-like defects resulting the Ce doped SnO_2_ behaviour as a p-type semiconductor^30^. On the other hand, it was essential to mention that above 3% Ce, the semiconducting behaviour transforms into p-type which has been one of the reason for the drastic increase in ethanol sensing. Analogous behaviour has been reported by Chattopahhyay and co-workers in case of Al doped SnO_2_^63^. It has been reported that below 12.05% Al substitution in SnO_2_, the sample exhibited n-type semiconducting behaviour, and it transforms to p-type above it. Aforementioned XPS results indicated that Ce^3+^ substitution at Sn^4+^ site leads to the formation of an acceptor-like defects in Ce doped SnO_2_ sample and introduced the excess amount of holes which in turn increases oxygen vacancies in the lattice^64–67^. Such increase in the defects can contribute well in the direction of achieving improved sensing to ethanol at RT. The highest sensor response noticed in 9 wt.% Ce-doped SnO_2_ is associated to increased oxygen vacancies in SnO_2_ lattice that provides active sites for adsorption of ethanol gas molecules at RT. Comprehending the statement that enhanced RT sensor response to ethanol in 9 wt.% Ce-doped sensors may result in formation of oxygen vacancies in the host materials. This conclusive statement is in agreement with ESR studies. From ESR studies, it is well-defined that oxygen vacancies are higher in case of 9 wt.% Ce-doped sensors. In addition, power absorption and peak to peak line width during resonance increases remarkably with Ce doping. Small ∆Hpp and highest intensity is noticed in 9% Ce doped SnO_2_ and resulted into the existence of large number of free electrons in the form of oxygen vacancies. Increase in sensitivity due to higher oxygen vacancies is previously reported in literature^68,69^. FESEM studies also confirmed that 9 wt % Ce doped SnO_2_ sensor exhibited the lowest grain size ~8 (2) nm and the number of grain boundaries increases by decreasing the gain size (see Supporting Information, Fig. S3). Therefore, surface areas increase steadily with the increased Ce concentration. Hence 9 wt % Ce doped SnO_2_ sensor shows a higher sensor response to ethanol at RT due to their larger surface areas. More surface areas provide more active sites for the adsorption of the chemical inputs which are responsible for the improvement of the gas sensor response. As discussed earlier from the TEM analysis, the variation in the particle size from 16 nm to 6 nm has been observed for the doping wt% ranging from 0 to 9% respectively. To understand the effect of particle size on the sensing mechanism, one needs to establish the relationship of particle size with Debye length depending on the type of metal oxide, the analyzed gas, and detection mechanism to be taken place^28^. The main fundamental parameter to evaluate the gas response is related to the Debye length (LD). There may be three cases to be assumed: (i) *d* ≫ *2Ls* (ii) *d* ~ *2Ls* (iii) *d* < *2Ls*. From the literature, it is considered that LD of SnO_2_ is about ~3 nm. In case of 0%, 3% and 6% Ce-SnO_2_, assumption 1 has been satisfied stating that the gas sensitivity may be practically independent of d because of the conduction mechanism taking place across the grain boundary is limited by Schottky barriers. But in case of 9% Ce-SnO_2_, the particle size becomes twice of the Debye length i.e. *d* ~ *2Ls*. This shows that the conductive channels between grains are overlapped attributed to the complete depletion of space charge region while interacting with the oxygen molecules. Hence, whole particle takes part in the gas sensing and as a result higher sensitivity is observed. So, the major factor for the higher sensing are attributed to the increased concentration of adsorbed oxygen species on the fully depleted junctions across the grains resulting into the higher sensing for ethanol at RT. In Table 3, the ethanol sensing response of the 9 wt% Ce doped SnO_2_ sensors are compared with those of the earlier published reports^70–82^. Table 3 displays marterials used, deposition technique, operating temperatures, sensor response, and recovery times of some ethanol sensors. However, sensor response of SnO_2_ based sensors are good, but operating temperatures are very high. On the other hand, it must also be observed from the present study that the 9 wt% Ce doped SnO_2_ sensor remarkably enhanced the ethanol sensor response at RT with faster response (recovery) time ~5–20 sec (~30–60 sec). Hence, the strikingly improved sensor response of 9% Ce-doped SnO_2_ sensor upon exposure of C_2_H_5_OH is associated to the uniformly distributed nanoparticles with size approximately twice of the Debye length, high surface area, defect chemistry and presence of a large number of unpaired electrons of the sensor surface.

**Table 3:** Comparison of the ethanol sensing capability of 9 wt% Ce doped SnO_2_ sensor in this study to those of the previous reports.

Materials Used	Deposition Technique	Operating temperature	Sensor Response	Response Time (Sec)	Recovery Time (Sec)	Reference
50 vol.% SnO_2_-ZnO	Combinational solution	300	4.69	72	NA	^70^
50 wt.% SnO_2_-ZnO	Electrospinning	360	17	5	1	^71^
5 wt.% La_2_O_3_-SnO_2_	Powder solution coating	300	740	1200	1200	^72^
ZnSnO_3_@SnO_2_	Hydrothermal	270	27.8	1	1.8	^73^
1.5 mol% Fe_2_O_3_-SnO_2_	Sol-gel	250	24	NA	NA	^74^
ZnO	Solvothermal	320	9.7	35	37.1	^75^
ZnO	Alkaline precipitation	300	275	36	35	^76^
ZnO/SnO_2_	Electrospinning	300	23	150	1100	^77^
MoO_3_	Exfoliation	300	33	21	10	^78^
In_2_O_3_/CuO	Sputtering	250	9.6	48	30	^79^
ZnO/SnO_2_	Hydrothermal	200	380	74	12	^80^
WO_3_	Hydrothermal	200	11.3	8.5	65	^81^
ZnO/SnO_2_	Hydrothermal	150	14.7	10	23	^82^
9 wt.% Ce doped SnO_2_	Drop casting	*RT*	382	~5–20	~30–60	***This work***

## Conclusions

In conclusion, systematic studies have been performed on the synthesis, characterization and RT gas sensing behaviour of p-type Ce doped SnO_2_ sensor. Pure and Ce doped SnO_2_ sensor have been successfully synthesized by chemical co-precipitation method and used to detect ethanol at RT. To the best of our knowledge, this is the first time a p-type Ce-doped SnO_2_ has been studied and demonstrated to meet the requirements for highly sensitive, selective and long-time stable low cost sensing device. The maximum sensitivity is ~382 for 400 ppm with fast response time of ~5 to 25 sec have been obtained in 9% Ce doped SnO_2_ towards ethanol at RT. The high sensitivity of Ce doped SnO_2_ sensor towards C_2_H_5_OH is attributed to the uniformly distributed nanoparticles with size approximately twice of the Debye length, defect chemistry, presence of a large number of unpaired electrons, and creating the excess of holes, as oxygen vacancies. This study suggests a practical approach to the fabrication and design of high performance ethanol sensors working at RT.

## Experimental

### Materials and Reagents

Tin(IV) chloride pentahydrate (SnCl_4_.5H_2_O), cerium(III) Chloride heptahydrate (CeCl_3_·7H_2_O), and stearic acid were purchased from Sigma-Aldrich. Ethanol (C_2_H_5_OH) and ammonia were purchased from Merck limited. All reagents were used without further purification.

### Synthesis

In present work, pure and Ce doped SnO_2_ samples are synthesized with three different Ce concentrations (the mole ratio of Ce/Sn = 3, 6 and 9 wt%) through a chemical co-precipitation method. Ethanol is used as a solvent for the synthesis of metal oxide nanoparticles. In a typical synthesis of Ce doped SnO_2_ sensor, SnCl_4_.5H_2_O and CeCl_3_.7H_2_O in stoichiometric proportions were dissolved in ethanol (C_2_H_5_OH). For the precipitation of the solution, ammonia and stearic acid was added drop wise to the precursor solution with continuous stirring and the final pH was maintained at 10. Obtained precipitates were filtered and washed several times with absolute alcohol and distilled water, then dried in a vacuum oven at 95 °C for 4 hours. These obtained powder samples (undoped and Ce doped SnO_2_) were annealed at 800 °C for six hours using a muffle furnace and were cool down at the rate of 100 °C per hour. The obtained powder samples have been used to fabricate the sensor device. The gold interdigital six finger electrodes patterns were deposited on the alumina substrates by thermal evaporation. The width of each finger is 500 µm and the interspacing between the fingers is 700 µm respectively. Then, the gas sensor was fabricated by drop casting technique. The pure and Ce-doped SnO_2_ dispersed in appropriate amount of ethanol followed by ultrsonication for 30 minutes. Thereafter, the solution was drop casted on the Au interdigital electrodes patterned substrate. The deposited sensor films were sintered at 95 °C for 30 minutes in a vacuum oven.

### Characterizations

The samples were characterized using X-ray diffraction (XRD, Bruker D8 advanced diffractometer) in the scanning range of 20–80° (2θ) with the step size of 0.0195° using Cu-Kα radiations at 1.54 Ǻ wavelength. The Rietveld refinement of XRD data has been done by FULLPROF program. Surface morphology and chemical composition of sensor materials were determined using field emission scanning electron microscope (FESEM, Model Σigma, Carl Ziess). The morphological and structural information were carried out using high-resolution transmission electron microscope (TEM, FEI Technai G230, Hillsboro, U.S.A.). To study the presence of unpaired electrons, electron spin resonance (ESR) spectra was recorded at RT by using ESR X-band spectrometer operating at 9.45 GHz frequency, 2.99 mW power with the sweeping time of 2 mins. X-ray photoelectron spectroscopy XPS (Perkin Elmer-1257 with hemispherical section analyzer with 25 meV resolution) has been used. XPS set up consist of dual anode X-ray source, which is capable of generating Al Kα (1486.6 eV) or Mg Kα (1253.6 eV) X-ray radiations. The sensing measurements were performed in custom designed gas sensing set up attached with mass flow controllers for precise measurement of gas flow at ppm level^39,83^.

## Electronic supplementary material

Supplementary information
