# Supplementary material for: New insights towards strikingly improved room temperature ethanol sensing properties of p-type Ce-doped SnO2 sensors
Source: Sci Rep. 2018 May 24;8:8079. doi: 10.1038/s41598-018-26504-3 (PMC5967327; doi:10.1038/s41598-018-26504-3)
Supplement: Supplementary file 1 — Supplementary information [file 41598_2018_26504_MOESM1_ESM.docx]

**Electronic Supplementary Material**

**New insights towards strikingly improved room temperature ethanol sensing properties of p-type Ce-doped SnO_2_ sensors**

Manjeet Kumar^1^, Vishwa Bhatt^1^, A. C. Abhyankar*^2^, Joondong Kim^1^, Akshay Kumar^3^, Sagar H. Patil^4^ and Ju-Hyung Yun*^1^

^1^Department of Electrical Engineering, Incheon National University, Incheon 406772, South Korea

^2^Department of Materials Engineering, Defence Institute of Advanced Technology, Girinagar, Pune 411025, India

^3^Department of Nanotechnology, Sri Guru Granth Sahib World University, Fatehgarh Sahib-140 407, Punjab, India

^4^Center for material characterization, CSIR-National chemical laboratory, Pashan, Pune-411008, India

*Corresponding Author Address:

Dr. Ju-Hyung Yun, E-mail: [juhyungyun@inu.ac.kr](mailto:juhyungyun@inu.ac.kr) , Phone: +82-32-835-8435.

Dr. A. C. Abhyankar, E-mail: [ashutoshabhyankar@gmail.com](mailto:ashutoshabhyankar@gmail.com) , Phone.: +91-20-24304311

**XRD analysis**

**

**

**Fig. S1:** Lattice parameters vs. Ce content in SnO_2_.

The lattice-strain developed in the samples of pure and Ce doped SnO_2_ nanomaterial was calculated by the Williamson-Hall equation.

βhklcosθ = ε (4sinθ) + λ/D (1)

where β is the FWHM of the diffraction peaks, θ is the Bragg angle, ε is the micro strain, λ is the wavelength of X-ray and D is the crystallite size. The Williamson-Hall plot of pure and Ce doped SnO_2_ nanomaterial samples are shown in Fig. S2.The micro strains of pure and Ce doped SnO_2_ nanomaterial samples were calculated from the slope of the plot between β cosθ and 4sinθ.


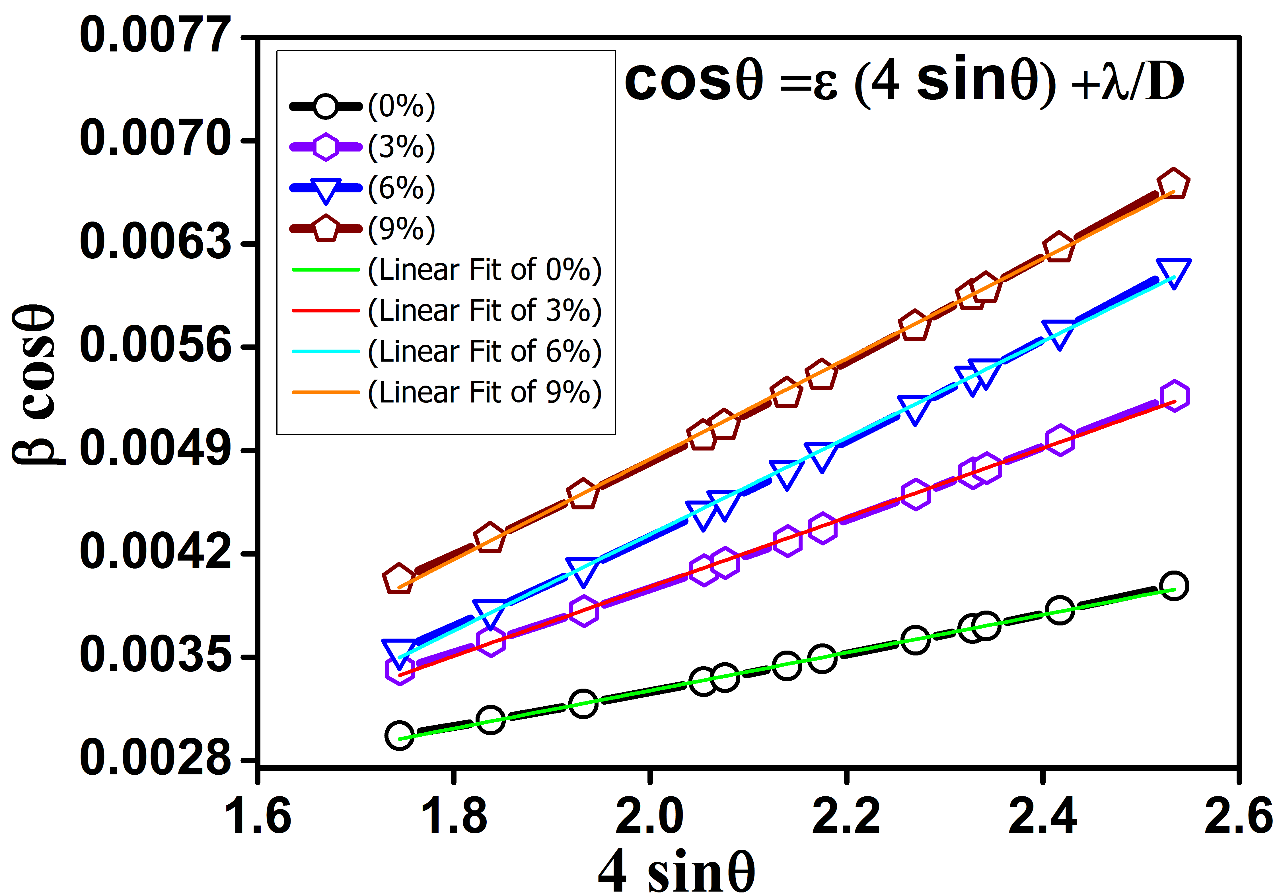


Fig.S2: The micro-strain calculated by the Williamson-Hall equation for pure and Ce doped SnO_2_ nanomaterial with addition of different concentrations of Ce: (0%, 3 %, 6 % and 9 %).

**FESEM analysis**

The surface morphology of the sensors material has been studied using FESEM. Fig. S3 shows FESEM images of pure and Ce doped SnO_2_ nanomaterial. Uniform distribution of particle size is observed in all the samples. Particle size is found to decrease with addition of Ce content in SnO_2_. The average grain size of pure and Ce doped SnO_2_ (0, 3, 6 and 9%) samples were observed ~20(5) nm, ~16(3) nm, ~14(2) nm and ~8(2) nm respectively. It is also observed that particles are spherical shape of pure and Ce doped SnO_2_ samples.EDS spectrum of pure and Ce doped SnO_2_ nanomaterial are shown in Fig. S4. The result clearly shows that there is no other impurity phase present in all the samples. The elemental composition for pure and Ce doped SnO_2_ is shown tabular form in inset of Fig. S4. The small variation in the actual composition have been observed in 3 and 9 % Ce doped SnO_2_ nanomaterial. The elemental distribution of the pure and Ce doped SnO_2_ nanomaterial was mapped using EDS by displaying the integrated intensity of Sn, O, and Ce shown in inset of Fig. S4. The elemental mapping shows the homogeneous distribution of each element in all the samples.


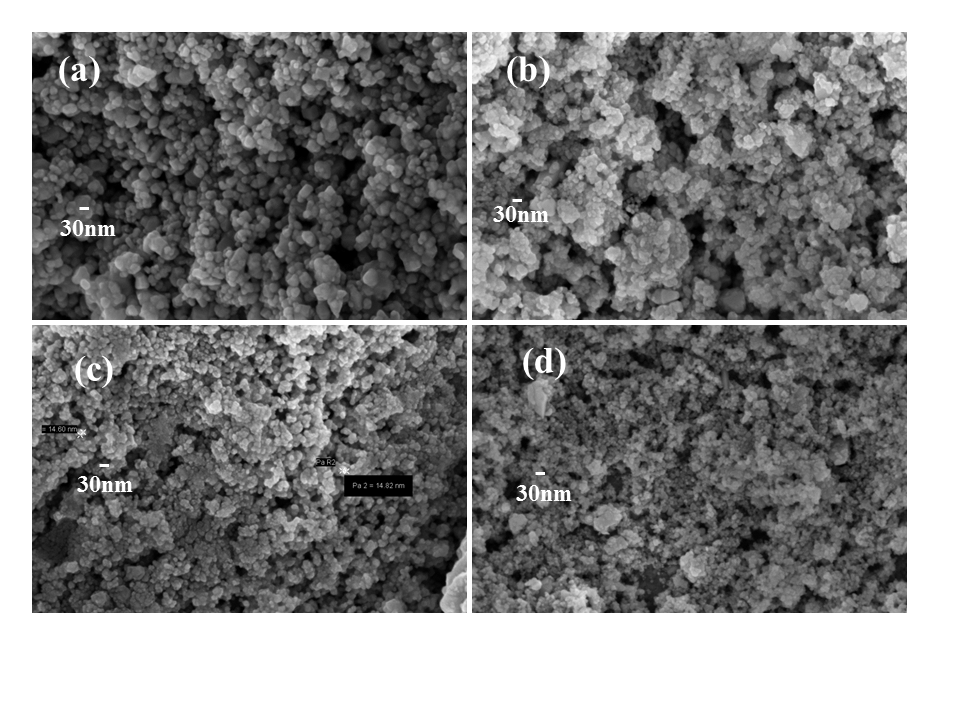


**Fig.S3:** FESEM micrograph of pure and Ce doped SnO_2_ sensor with addition of different concentrations of Ce: (a) 0%, (b) 3 %, (c) 6 % and (d) 9 %.


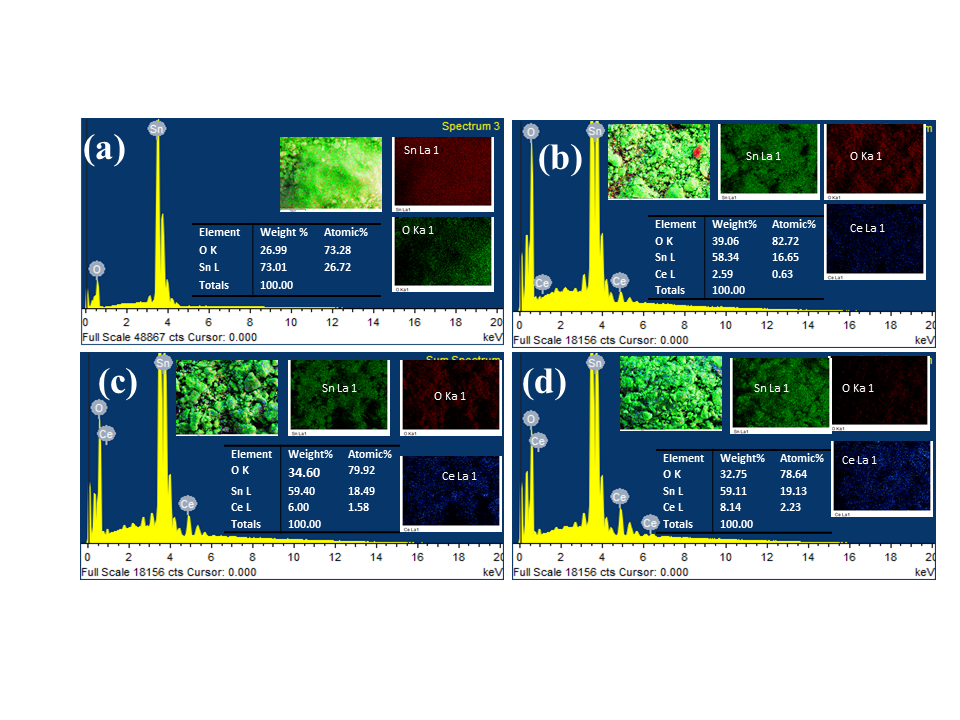


**Fig.S4:** The EDAX spectra of pure and Ce doped SnO_2_ with addition of different concentrations of Ce: (a) 0%, (b) 3 %, (c) 6 % and (d) 9 %. Inset shows elemental distribution mapping & elemental composition data.

**Sensing measurements**


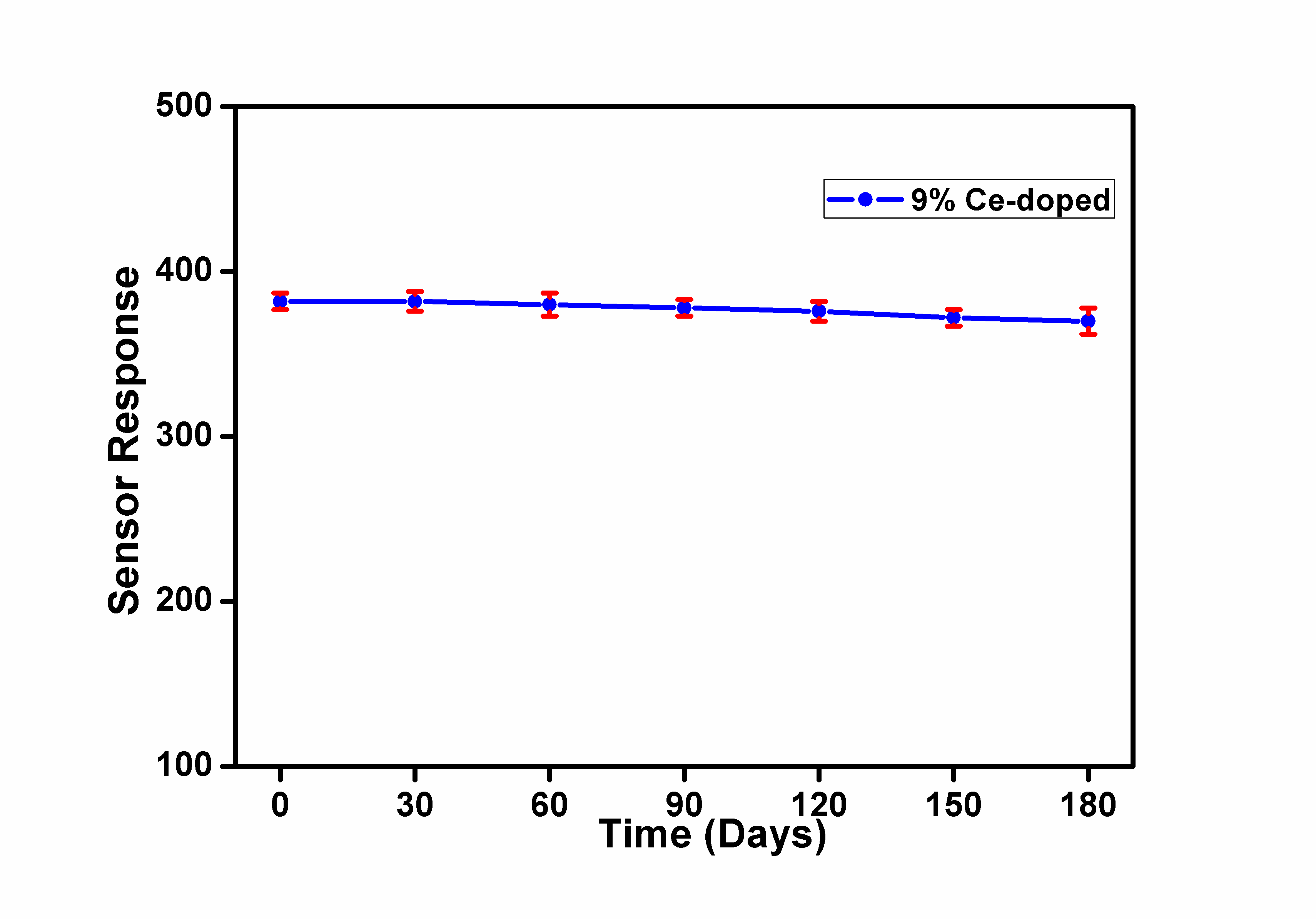


**Fig. S5:** The long-term stability response of the 9 % Ce doped sensor towards 400 ppm ethanol exposure at RT.

**Table S1:** Average parameters calculated from Hall effect measurements for pure and Ce doped SnO_2_

| Sample | Bulk Concentration (cm^-3^) | Mobility (cm^2^/V.s) | Avg. Hall co-efficient(cm^2^/C) | Carrier type |
| --- | --- | --- | --- | --- |
| Pure SnO_2_ | -2.83E+13 | 5.35E+00 | -2.21E+05 | n |
| 3 wt. % Ce | 3.99E+13 | 5.69E-01 | 1.57E+05 | p |
| 6 wt.% Ce | 7.35E+13 | 1.96E-01 | 8.49E+04 | p |
| 9 wt.% Ce | 2.26E+14 | 7.46E-02 | 2.76E+04 | p |

Hall measurements were carried out by Ecopia Corporation, HMS-5000 using the van der Pauw contact configuration. For each sample, Hall measurement has been taken for 20 times & average value of Hall parameters measured for pure and Ce doped SnO_2_ samples are tabulated in table S1. The Hall measurement results tabulated in table S1 indicate that the hole concentration increases along with increasing Ce concentration up to 9 wt. % in SnO_2_ lattice. The 9 wt. % Ce doping exhibits high hole concentration (2.26 × 10^14^ cm^−3^) and low mobility (7.46E-02 cm^2^/V.s) as compared to other samples. From the table, it has been confirmed that the hall coefficient is negative for pure SnO_2_ and found to be positive for Ce doped samples which confirms the p-type behavior of Ce doped SnO_2_ samples.
